# Supplementary material for: Amphiphilic Cationic Peptide-Coated PHA Nanosphere as an Efficient Vector for Multiple-Drug Delivery
Source: Nanomaterials (Basel). 2022 Aug 31;12(17):3024. doi: 10.3390/nano12173024 (PMC9457696; doi:10.3390/nano12173024)
Supplement: Supplementary file 1 [file nanomaterials-12-03024-s001.zip › nanomaterials-1853464-supplementary.pdf]

Supplementary Materials

Amphiphilic Cationic Peptide-Coated PHA Nanosphere as an Efficient Vector for Multiple-Drug Delivery

Fanghua Zhang [1,3], Chao Zhang [2], Shuangqing Fu [1], Huandi Liu [1], Mengnan Han [1], Xueyu Fan [1], Honglei Zhang [1,*] and Wei Li [1,*]

1 College of Chemistry and Environmental Science, Key Laboratory of Chemical Biology of Hebei Province, Laboratory of Medicinal Chemistry and Molecular Diagnosis of the Ministry of Education, Institute of Life Science and Green Development, Hebei University, Baoding 071002, China
2 Department of Life Science, Hengshui University, Hengshui 053000, China
3 Tianjin Key Laboratory of Molecular Optoelectronic Sciences, Department of Chemistry, School of Science, Tianjin University, Tianjin 300072, China
* Correspondence: zhanghonglei@hbu.edu.cn (H.Z.); liwei2020@hbu.edu.cn (W.L.)

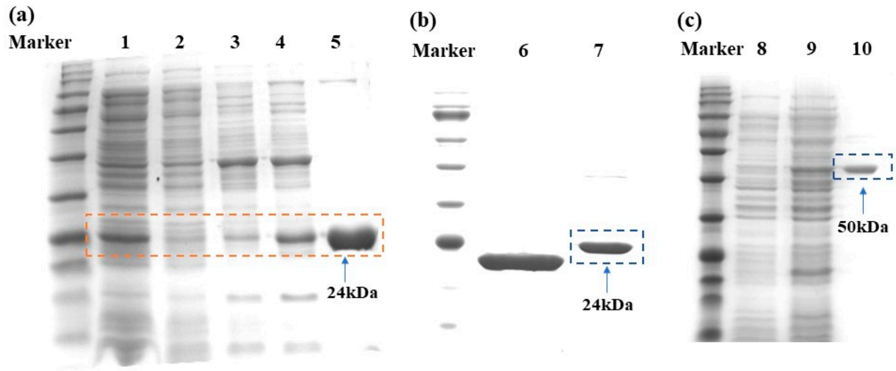

Figure S1. SDS polyacrylamide gel electrophoresis. (a) 1-5: gradient elution of PR protein with eluent containing 20, 40, 60, 80 and 500 mM imidazole. (b) 6 - 7: purified PhaP protein and fusion protein. (c) 8-10: recombinant *E. coli* BL21 (DE3)/ *phap-egfp* induced without IPTG, the crushing supernatant of recombinant *E. coli* BL21 (DE3) / *phap-egfp* induced by IPTG, and purified fusion protein PE.

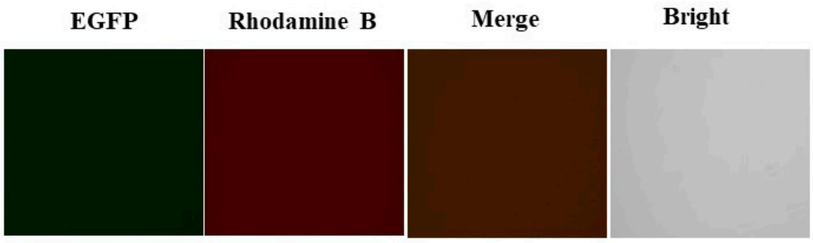

Figure S2. Laser confocal images of Rho@PHBX and PE unassembled.

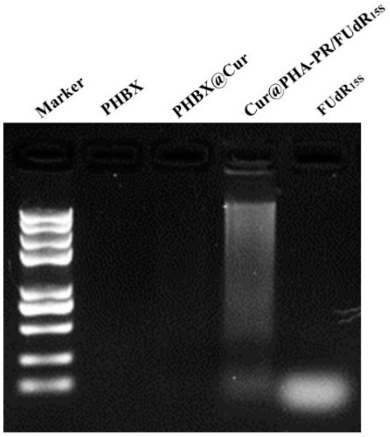

Figure S3. Analysis of PHBX, PHBX@Cur, Cur@PHBX-PR/FUdR15S and FUdR15S by agarose electrophoresis.

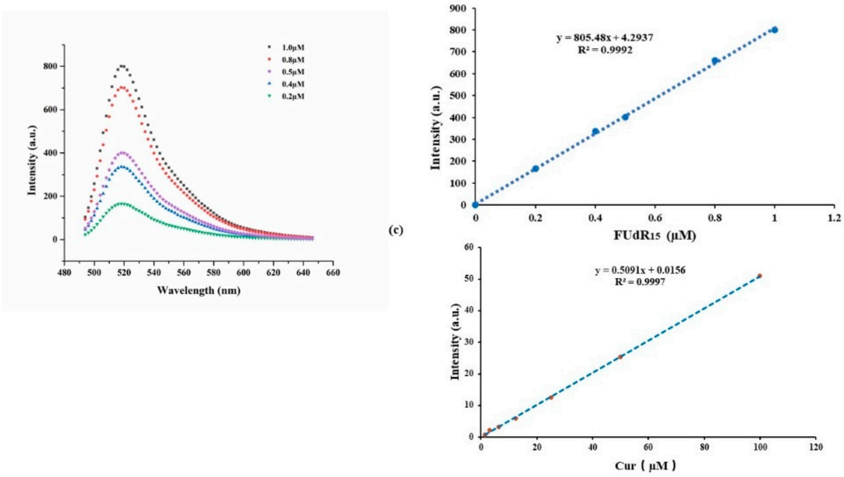

Figure S4. Fluorescence spectrum of FUdR15S (a), standard curve graph of FUdR15S (b) and Cur (c).

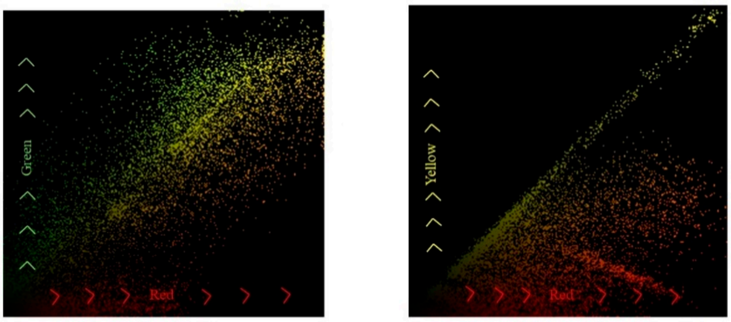

Figure S5. The co-localization of FUdR and Cur in lysosomes. Tracing was performed using a lysosomal locator, and data analysis was conducted using image pro plus software (Cur@PHBX-PR/FUdR15S was Green and the lysosomes was Red).

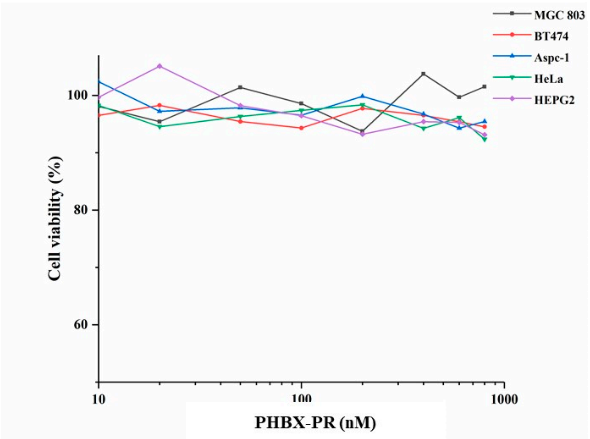

Figure S6. The cytotoxicity of cancer cells treated with PHA-PR.

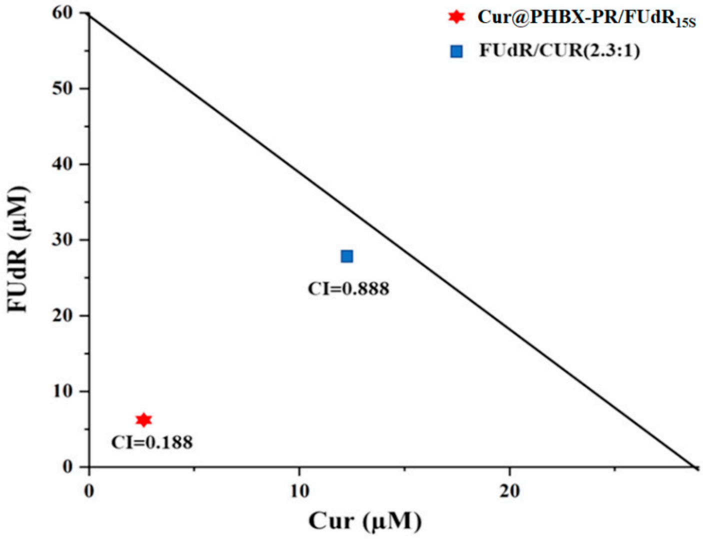

Figure S7. The analysis of the synergetic effect of FUdR and Cur.

Table S1. IC50 values of FUdR and Cur in MGC 803 cells.

Name	FUdR (μM)	Cur (μM)	IC$_{50}$ FUdR/Cur(2.3:1) FUdR (μM))	FUdR/Cur(2.3:1) Cur (μM)	Cur@PHBX-PR/FUdR$_{15S}$ FUdR (μM))	Cur@PHBX-PR/FUdR$_{15S}$ Cur (μM)
**MGC 803**	**60.04±2.431**	29.03±3.275	27.63±3.328	12.42±3.354	15.964±3.560	2.575±3.509
BT474	121.4±2.374	39.55±2.729	48.22±3.399	20.96±3.414	25.54±4.308	11.11±4.334
HeLa	62.46±2.648	43.22±4.085	28.39±2.946	12.36±2.958	21.83±4.486	9.506±4.506
Aspc-1	75.83±5.418	51.30±3.743	33.93±3.830	14.76±3.849	19.77±4.219	8.609±4.206
HEPG2	95.81±3.666	56.5±2.879	58.91±5.098	25.62±5.096	40.41±2.902	17.58±2.904
